# Intravenous iron delivers a sustained (8‐week) lowering of pulmonary artery pressure during exercise in healthy older humans

**DOI:** 10.14814/phy2.14164

**Published:** 2019-07-03

**Authors:** Hung‐Yuan Cheng, Matthew C. Frise, M. Kate Curtis, Nicole K. Bart, Nayia Petousi, Nick P. Talbot, George M. Balanos, Peter A. Robbins, Keith L. Dorrington

**Affiliations:** ^1^ Department of Physiology, Anatomy & Genetics University of Oxford Oxford United Kingdom; ^2^ School of Sport, Exercise and Rehabilitation Sciences University of Birmingham Birmingham United Kingdom

**Keywords:** Exercise, iron, pulmonary circulation, pulmonary hypertension

## Abstract

In older individuals, pulmonary artery pressure rises markedly during exercise, probably due in part to increased pulmonary vascular resistance and in part to an increase in left‐heart filling pressure. Older individuals also show more marked pulmonary vascular response to hypoxia at rest. Treatment with intravenous iron reduces the rise in pulmonary artery pressure observed during hypoxia. Here, we test the hypothesis that intravenous iron administration may also attenuate the rise in pulmonary artery pressure with exercise in older individuals. In a randomized double‐blind placebo‐controlled physiology study in 32 healthy participants aged 50–80 years, we explored the hypothesis that iron administration would deliver a fall in systolic pulmonary artery pressure (SPAP) during moderate cycling exercise (20 min duration; increase in heart rate of 30 min^−1^) and a change in maximal cycling exercise capacity (${\dot{V}}_{O2max}$). Participants were studied before, and at 3 h to 8 weeks after, infusion. SPAP was measured using Doppler echocardiography. Iron administration resulted in marked changes in indices of iron homeostasis over 8 weeks, but no significant change in hemoglobin concentration or inflammatory markers. Resting SPAP was also unchanged, but SPAP during exercise was lower by ~3 mmHg in those receiving iron (*P* < 0.0001). This effect persisted for 8 weeks. Although ${\dot{V}}_{O2max}$ remained unaffected in the iron‐replete healthy participants studied here, this study demonstrates for the first time the ability of intravenous iron supplementation to reduce systolic pulmonary artery pressure during exercise.

## Introduction

Increasing age is associated with a reduction in exercise capacity. Although we do not find this surprising, the factors accounting for it are unclear. One potential contributory factor that has emerged in recent studies is the marked rise in pulmonary artery pressure that occurs during exercise in older people, which increases the workload of the right ventricle for any given cardiac output. In a review of 47 publications containing catheter data from 1187 subjects, Kovacs and colleagues found that mean pulmonary artery pressure (MPAP) at rest varied little with age, whereas during mild exercise it increased markedly with age (<30 years: to 18.2 ± 5.1 mmHg; 30–50 years: to 20.0 ± 4.7 mmHg; ≥50 years: to 29.4 ± 8.4 mmHg (*P* < 0.001)) (Kovacs et al. 2009a). Similar findings were reported by Mahjoub and colleagues using echocardiographic measurement of systolic pulmonary artery pressure (SPAP) over the age range 20–80 years and including both light and heavy exercise (Mahjoub et al. 2009).

An increase in pulmonary artery pressure can be attributed to an elevation in left atrial pressure at outflow from the lungs, an increase in the pressure drop across the pulmonary vessels themselves (and hence pulmonary vascular resistance), or to a combination of these two factors. A subsequent review by Kovacs and colleagues has dissected out these two components in 88 volunteers undergoing catheter studies divided into four age groups (Kovacs et al. 2012). These investigators found that pulmonary vascular resistance was higher in older people (>70 years) than in the youngest group (<24 years) by ~50% at all comparable levels of CO achieved during exercise. However, the most striking contributor to an elevation in MPAP in volunteers above 50 years of age was a large increase in pulmonary outflow pressure, measured as pulmonary artery wedge pressure, commonly rising to ~20 mmHg during light and heavy exercise. Of note in view of this observation is that two studies have suggested that older individuals also show an exaggerated pulmonary hypertensive response to acute hypoxia at rest (Balanos et al. 2015; Turner et al. 2015). Any strategy to lower MPAP during exercise needs to lower pulmonary vascular resistance, pulmonary artery wedge pressure or both. In this study we harness previous findings to attempt to reduce pulmonary artery pressure during exercise by targeting pulmonary vascular resistance.

Three considerations are brought to bear. The first is that exercise is associated with a marked desaturation of mixed venous blood as oxygen extraction increases up to approximately threefold in heavy exercise (Sutton et al. 1988; Trinity et al. 2012). Second, the elevation of pulmonary vascular resistance during exposure of the lung to hypoxia is known from many (non‐human) studies to be a response in part from a lowering of partial pressure of oxygen (P_O2_) in mixed venous blood arriving at the lungs as well as the lowering of the P_O2_ of alveolar gas (Marshall and Marshall 1983; Marshall et al. 1994aa). Third, the sensitivity with which this hypoxic pulmonary vasoconstriction responds to its hypoxic stimulus has been shown in several human studies to be ameliorated substantially by elevation of the level of iron in the body following intravenous supplementation with iron sucrose (Talbot et al. 2014) or ferric carboxymaltose (Smith et al. 2008; Bart et al. 2016, 2018; Frise et al. 2016). This sequence of connections raises the possibility that iron supplementation may offer a mechanism for reducing pulmonary vascular resistance, and therefore right‐ventricular workload, during euoxic exercise.

We hypothesized first that administration of ferric carboxymaltose to participants aged 50–80 years would reduce pulmonary artery pressure during exercise, and second that this would be reflected in an increase in maximal exercise capacity. We explored these hypotheses in a prospective randomized double‐blind placebo‐controlled clinical physiology study, which confirmed the first but not the second hypothesis.

## Methods

### Participants

Thirty‐two healthy men and women aged 50–80 years volunteered to participate in this study. Inclusion criteria were as follows: not a current smoker; body mass index (BMI) <30 kg/m^2^; no history of severe cardiac disease, respiratory diseases or epilepsy; receiving no iron supplements or medications affecting the pulmonary circulation during the previous 3 months; able to perform bicycling exercise. In addition, participants were required to have detectable tricuspid regurgitation on echocardiography for the estimation of SPAP. At a screening visit, measurements were made of forced expiratory volume in 1 sec (FEV_1_), forced vital capacity (FVC), systemic arterial blood pressure (Omron M7 Digital Blood Pressure Monitor, Omron, UK), and resting consumption of oxygen (${\dot{V}}_{O2}$) and elimination of carbon dioxide (${\dot{V}}_{CO2}$) in a manner described below. This study was approved by the National Research Ethics Service Committee South Central‐Oxford B (Reference: 11/SC/0221) and was performed in accordance with the general principles of the Declaration of Helsinki. All participants provided written informed consent.

### Experimental protocol

**Figure 1:**
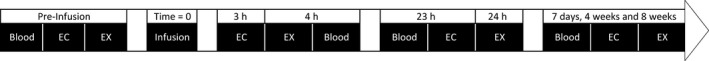
Schematic diagram of time course of the protocols. Blood: blood samples; EC: echocardiography at rest and during light cycle exercise; EX: ${\dot{V}}_{O2max}$ exercise. Infusion refers to the blinded administration of either ferric carboxymaltose or saline. For further details see text.

The study design is summarized in Figure 1. In short, physiological and hematological measurements were made immediately before double‐blind administration of an infusion of a single dose of either ferric carboxymaltose (15 mg/kg to a maximum of 1 g) or placebo saline, and on five occasions in the period of 8 weeks after the administration. Participants were randomized into iron and control groups, according to a block randomization strategy using groups of four, with males and females in separate groups. We aimed to induce measureable changes in iron status in participants who were allocated ferric carboxymaltose. We then observed in these, and their controls, SPAP and cardiac output during light exercise, and work rate and oxygen consumption during maximal exercise (${\dot{V}}_{O2max}$).

Participants arrived in the morning around 9 am for the first blood sample and exercise tests. After a short break (20–30 min), participants received an infusion over 15 min. The same tests were repeated at 3 h, 23 h, 7 days, 4 weeks, and 8 weeks after the infusion.

### Echocardiographic measurement of SPAP and cardiac output during light exercise

Light exercise was performed on a cycle ergometer (Ergoline Company, Germany) in the semi‐supine position with 30–40 degrees of rotation to the left. The exercise protocol included 5 min of rest, 20 min of exercise, and 5 min of a resting recovery phase. SPAP was estimated using measurements of peak systolic tricuspid regurgitant jet velocity (*V*_TR_), obtained from an apical 4‐chamber view of the heart using continuous wave Doppler, as 4V_TR_^2^ + 5 mmHg (Rudski et al. 2010) at rest and during a level of exercise that increased the heart rate to 30 bpm above the resting value, an increase known to be associated with 50% of maximum oxygen consumption (Herigstad et al. 2007). Cardiac output was measured using standard techniques by visualizing the left‐ventricular outflow tract via an apical 5‐chamber view of the heart, and integrating the outflow velocity from pulsed‐wave Doppler with respect to time over several heartbeats (Balanos et al. 2005; Talbot et al. 2005). The left‐ventricular outflow tract diameter was estimated from a parasternal long‐axis view. The baseline values of echocardiographic measurement were determined as the averaged echocardiographic data over the resting period. The exercise values of echocardiographic measurement were determined as the averaged echocardiographic data over the second half of the exercise period.

### Measurement of ${\dot{V}}_{O2\max}$ during an incremental exercise test

The incremental exercise test was performed on a cycle ergometer (KEM‐3, CardioKinetics.

Limited, UK) with a face mask (Metro SealTM 7900 Adult Face mask, Hans Rudolph Inc., USA) to determine ${\dot{V}}_{O2max}$ and maximum work rate using a metabolic measurement system (JaegerTM Oxycon^®^, Carefusion, UK). The exercise protocol was adapted from that described by Rossiter et al. (2006). Each participant performed a standard incremental exercise test to the point of volitional exhaustion with the work rate rising incrementally at 15 W per minute, from 0 W. At the point of exhaustion, participants were asked to continue pedaling at 0 W for a further 5 min, after which the work rate was increased abruptly to a value 105% of the maximum work rate achieved during the incremental test. The participant was again asked to pedal to exhaustion. During both phases of the protocol, exhaustion was determined by participants stopping or by the cadence falling below 60 rpm. The ${\dot{V}}_{O2max}$ was determined as the average of ${\dot{V}}_{O2}$ values over 15 sec during the period of maximum ${\dot{V}}_{O2}$ during the exercise test. If the highest ${\dot{V}}_{O2}$ values achieved differed between the two exhaustion periods, the higher value was taken to represent the participant's ${\dot{V}}_{O2max}$. Baseline ${\dot{V}}_{O2}$ and CO_2_ elimination (${\dot{V}}_{CO2}$) were taken as the average of measurements from the 2nd minute on the ergometer before pedaling commenced.

Peripheral oxyhemoglobin saturation (SpO_2_) was measured using a pulse oximeter (Ohmeda Biox 3740 Pulse Oximeter, BOC Healthcare, UK) with a finger probe (OXY‐F4‐H finger sensor, GE Healthcare, UK).

### Infusion of ferric carboxymaltose or control saline

The infusion procedure was double‐blind: volunteers wore eye masks during infusions, which were administered by physicians not involved in data analysis. Each participant in the iron group received 15 mg/kg of iron (up to a maximum dose of 1000 mg) as ferric carboxymaltose (Ferinject^®^, Vifor Pharma) diluted to 50 mL with 0.9% NaCl. The control infusion consisted of 50 mL 0.9% NaCl. The infusion time was 15 min and controlled by a syringe pump (GrasebyTM 3100 Syringe Pump, Smith Medical International Ltd, UK).

### Blood samples and analytical procedures

Venous blood samples were obtained from a forearm vein for assay of hemoglobin (Hb), hematocrit (Hct), mean cell volume (MCV), serum iron, serum ferritin, serum transferrin, transferrin saturation, plasma hepcidin, and plasma C‐reactive protein (CRP). Blood was distributed into tubes containing EDTA (EDTA BD Vacutainer^®^, BD) or micronized silica particles (SST BD Vacutainer^®^, BD) for immediate hematology and biochemistry analysis; plasma was prepared by centrifugation at 3000*g* and immediately frozen at –80°C for later analysis of hepcidin by enzyme‐linked immunosorbent assay (ELISA, Hepcidin‐25 human‐ EIA Kit, Bachem). The ELISA analysis was in duplicate and followed the guidelines of the manufacturer.

If a participant's CRP was elevated above the normal range for the assay (>8 mg/L), hematological indices from that visit were excluded from analysis in case there was an intercurrent mild inflammatory illness that might have temporarily disturbed iron homeostasis (Feelders et al., 1998; Gabay and Kushner, 1999; Ganz, 2003).

### Statistical analysis

Between‐groups comparisons of participant characteristics were performed using a two‐tailed Student's *t*‐test. To assess changes in blood and iron indices and changes in hemodynamic variables after infusion, a two‐way repeated measures analysis of variance (ANOVA), using time and group as factors, was performed using SigmaPlot (Version 12.0, Systat Software). The Bonferroni procedure was used in post hoc analysis to perform the multiple comparison. Results are given as mean ± SEM if not otherwise stated.

To assess the effect of iron infusion on SPAP, ${\dot{V}}_{O2max}$ and maximum work rate, results were analysed using linear mixed effects modeling (SPSS version 23), with the model structures as indicated in the Results section. In all cases, *P* less than 0.05 was taken to be statistically significant.

## Results

### Participant characteristics

The characteristics of enrolled participants are shown in Table 1. There was no significant difference between groups with regard to these characteristics. The infusions were well tolerated with no severe adverse reactions or withdrawals. Two participants (one in each group) experienced headache after the infusion and one participant from the iron group reported a fever the day after the infusion.

**Table 1:** Participant characteristics. Values are shown as mean ± SD

	Iron group	Control group
Number, *n*	16	16
Sex, %male	50%	50%
Age, year	65.4 ± 8.6	64.6 ± 4.9
Height, cm	167.5 ± 10.1	168.0 ± 8.5
Weight, kg	67.3 ± 14.8	67.9 ± 11.0
FEV_1_, % predicted	108.1 ± 17.1	107.1 ± 16.9
FVC, % predicted	117.6 ± 16.5	111.9 ± 14.0
FEV_1_/FVC	74.2 ± 8.8	76.2 ± 6.0
Systolic blood pressure, mmHg	124.4 ± 13.7	121.6 ± 15.4
Diastolic blood pressure, mmHg	80.0 ± 8.3	81.9 ± 10.9
Baseline ${\dot{V}}_{O2}$, mL/min	299 ± 18	308 ± 17
Baseline ${\dot{V}}_{CO2}$, mL/min	239 ± 17	239 ± 12

### Iron status indicators and hematological variables

**Figure 2:**
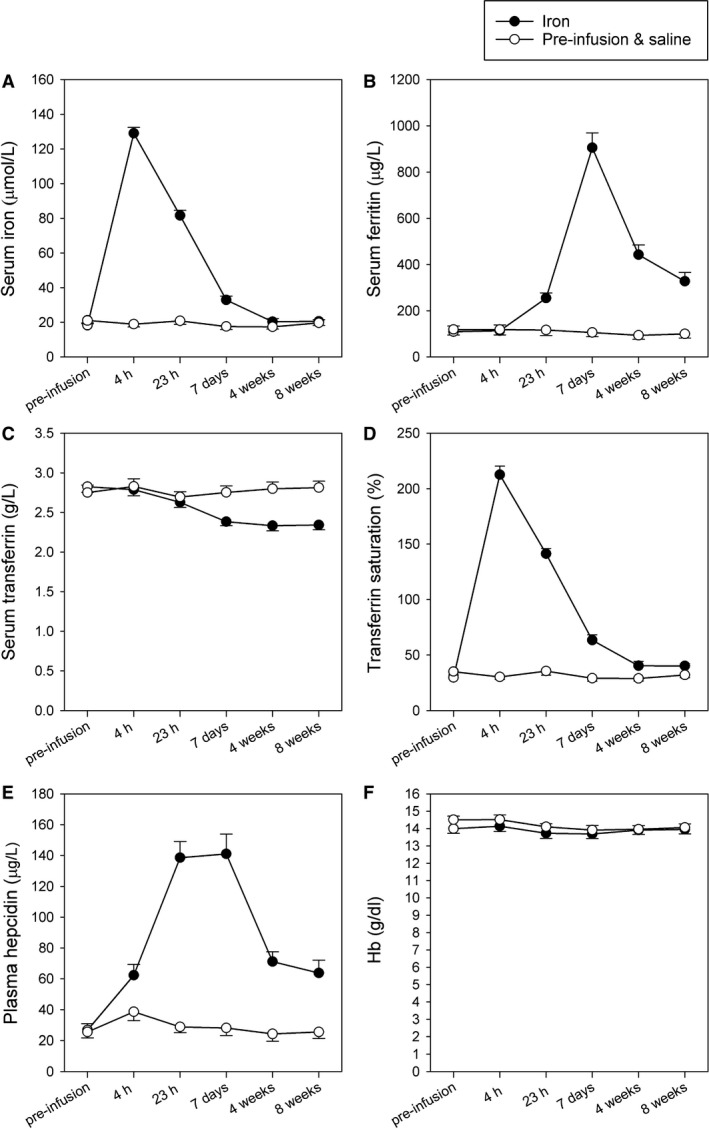
Iron indices and hemoglobin concentration. A. Serum iron concentration; B. Serum ferritin concentration; C Serum transferrin concentration; D Transferrin saturation; E. Plasma hepcidin concentration; F. Hemoglobin (Hb) concentration. Empty circles represent data before infusions and the control group throughout. Filled circles represent data following iron administration in the iron group. Values are mean ± SEM.

At baseline, the iron and the placebo groups had similar iron status as indicated by concentrations of serum iron, serum ferritin, serum transferrin, transferrin saturation, and plasma hepcidin (Fig. 2). After the infusion, a rapid significant increase in serum iron was seen in the iron group. The highest iron concentration was reached at 4 h and dropped to reach the control value 4 weeks after the infusion (Fig. 2A). The ferritin and hepcidin concentrations following iron infusion rose substantially, respectively, by 23 and 4 h, and stayed significantly raised throughout the 8 weeks (Fig. 2B and E). The transferrin concentration in the iron group fell by 23 h and remained low for the 8‐week period (Fig. 2C). Transferrin saturation followed the pattern of change of serum iron (Fig. 2D), values above 100% indicating short‐term presence of free iron in plasma and/or iron bound to species other than transferrin (Zanen et al. 1996). In contrast, concentrations of iron, ferritin, transferrin, transferrin saturation, and hepcidin in the control group remained unchanged. These data show that iron was successfully delivered to the circulation and led to a long‐term rise in ferritin concentration consistent with the dosage administered (Bart et al. 2018).

On ten occasions (iron group 7; placebo group 3), CRP exceeded 8 mg/L and laboratory blood‐test data for these time points were excluded from the analysis. A sensitivity analysis exploring the effect of including these data found no significant alteration to the reported findings.

At baseline, the iron and the placebo groups showed no significant difference with regard to hemoglobin concentration (Fig. 2F), Hct, or MCV. No significant changes were seen in hemoglobin concentration in either group throughout the 8 weeks (Fig. 2F). No significant changes in Hct or MCV were seen at any time point following iron or saline infusion.

### Cardiovascular variables at rest and during light exercise

At baseline, in the resting condition, no significant differences were measured between the iron and placebo groups with respect to any cardiovascular variable.

**Figure 3:**
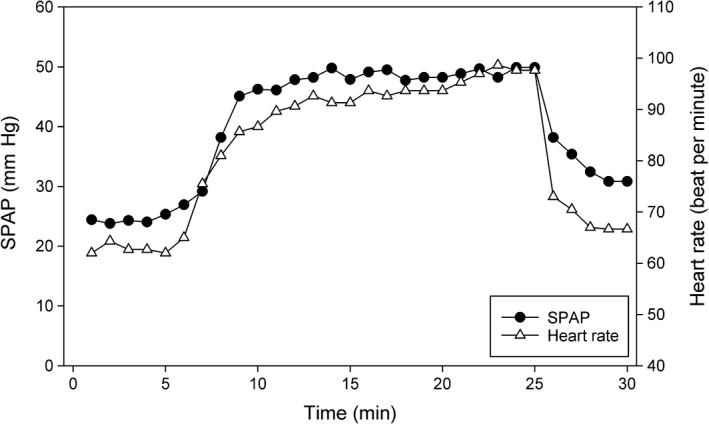
Acquisition of echocardiographic data during a light cycle exercise test from a representative participant. The participant rested on the ergometer for 5 min and then started exercise aiming to reach a target heart rate of 30 bpm above resting heart rate (empty triangles). Exercise was sustained for 20 min and the participant was then allowed to rest. Systolic pulmonary artery pressure (SPAP, black circles) was measured for the whole duration. Data obtained from first 5 min were averaged and taken as the resting value. Data between 16th and 25th minute were averaged and taken as the exercise value.

Figure 3 shows the time‐course of SPAP and heart rate over 30 min for a typical volunteer undergoing the moderate exercise protocol. This protocol stipulated a rise in heart rate of 30 bpm from rest to light exercise. An analysis of the mean heart rate achieved over all time points showed close compliance with the protocol in both groups (change in the iron group 29.0 ± 0.3 bpm; change in the placebo group 28.5 ± 0.6 bpm (*P *=* *0.46)). Table 2 presents cardiac output and SPAP pre‐infusion and at five times in the 8‐week period following infusion. At baseline, during exercise, as during rest, no significant differences were measured between the iron and saline groups with respect to any cardiovascular variable.

**Table 2:** Hemodynamic data preinfusion and at five times in the 8‐week period following infusion. Values are shown as mean ± SEM

	Preinfusion	3 h	23 h	7 days	4 weeks	8 weeks
CO at rest with placebo, L/min	4.46 ± 0.15	5.06 ± 0.22^##^	4.93 ± 0.13^a^	4.80 ± 0.14	5.07 ± 0.23^##^	4.70 ± 0.15
CO in exercise with placebo, L/min	7.60 ± 0.19	8.09 ± 0.24	7.75 ± 0.18	7.70 ± 0.21	7.73 ± 0.26	7.99 ± 0.18
CO at rest with iron, L/min	4.48 ± 0.20	4.85 ± 0.23	4.76 ± 0.22	4.99 ± 0.20**	4.71 ± 0.21	4.82 ± 0.16
CO in exercise with iron, L/min	7.65 ± 0.31	7.90 ± 0.32	7.94 ± 0.34	7.94 ± 0.31	7.90 ± 0.28	7.96 ± 0.25
SPAP at rest with placebo, mmHg	24.8 ± 1.0	25.0 ± 0.9	25.0 ± 1.0	24.9 ± 1.00	25.5 ± 0.9	24.7 ± 0.9
SPAP in exercise with placebo, mmHg	38.9 ± 1.8	39.0 ± 1.7	38.9 ± 1.9	38.8 ± 1.9	39.0 ± 1.7	38.5 ± 1.8
SPAP at rest with iron, mmHg	23.0 ± 0.7	22.9 ± 0.7	23.5 ± 0.7	23.4 ± 0.8	23.3 ± 0.6	22.7 ± 0.7
SPAP in exercise with iron, mmHg	39.4 ± 1.0	38.6 ± 0.9	37.2 ± 0.9^***^	37.1 ± 1.0^***^	37.2 ± 0.9^***^	36.9 ± 0.9^***^

a Different from the preinfusion measurement in the placebo group: ^#^ *P* < 0.05, ^##^ *P* < 0.01.

Different from the preinfusion measurement in the iron group: **P < 0.01, ***P < 0.001.

**Figure 4:**
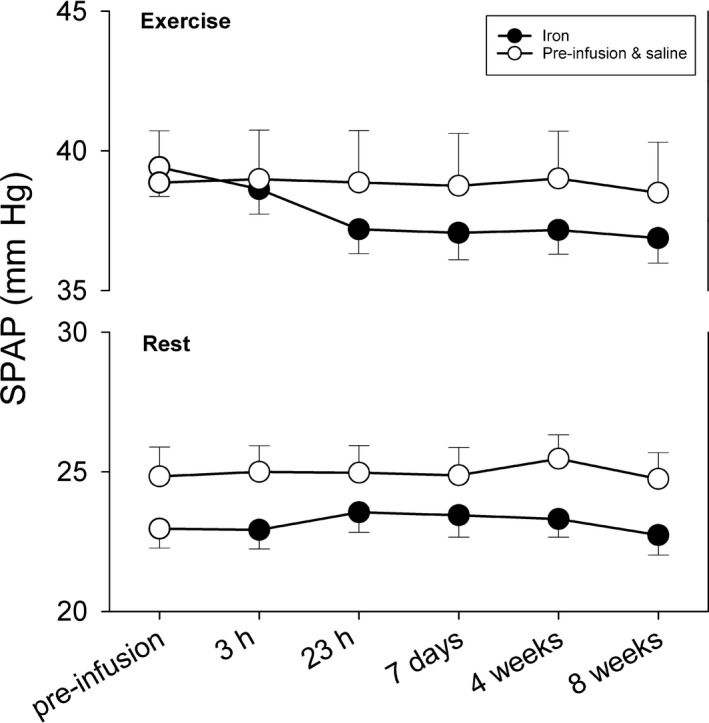
Systolic pulmonary artery pressure (SPAP) at rest and during moderate exercise. Empty circles represent data before infusions and the control group throughout. Filled circles represent data following iron administration in the iron group. Values are mean ± SEM. Iron administration lowers SPAP significantly during exercise (*P* < 0.0001) but not at rest.

**Figure 5:**
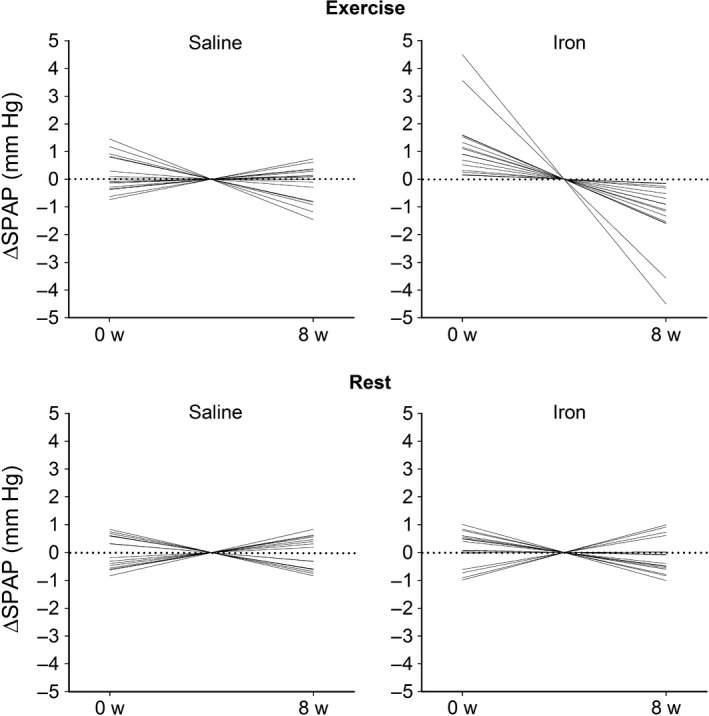
Individual changes in systolic pulmonary artery pressure (ΔSPAP) at rest and during exercise from the preinfusion values (0 week) to the 8‐week values (8 weeks) normalized to be zero mid experiment. In those receiving iron, each individual participant showed a decline in exercise SPAP during this period. No consistent change occurred in the three other conditions.

Figure 4 shows SPAP at rest and during moderate exercise for the group receiving iron and the controls. Linear mixed effects modeling on the postinfusion data using the preinfusion pressure as a covariate and with fixed factors of treatment (iron vs. saline) and exercise‐by‐treatment revealed a significant effect of exercise‐by‐treatment (*P *<* *0.0001). Successive data points for each subject were treated as repeated measures. Subgroup analysis on the rest and exercise data separately revealed that treatment had a significant effect only on the exercise data. It is notable from Figure 4 that the effect of iron during exercise persisted for the whole of the 8‐week period. Figure 5 shows the individual changes in SPAP at rest and during exercise from the pre‐infusion values to the 8‐week values normalized to be zero mid‐experiment. In those receiving iron, each individual participant showed a decline in exercise SPAP during this period. No consistent change occurred at rest in those receiving iron. In the control group, no consistent change is seen, either at rest or during exercise, during this period of 8 weeks.

*V* ${\dot{V}}_{O2max}$, work rate, and SpO_2_ during the incremental exercise test.

**Figure 6:**
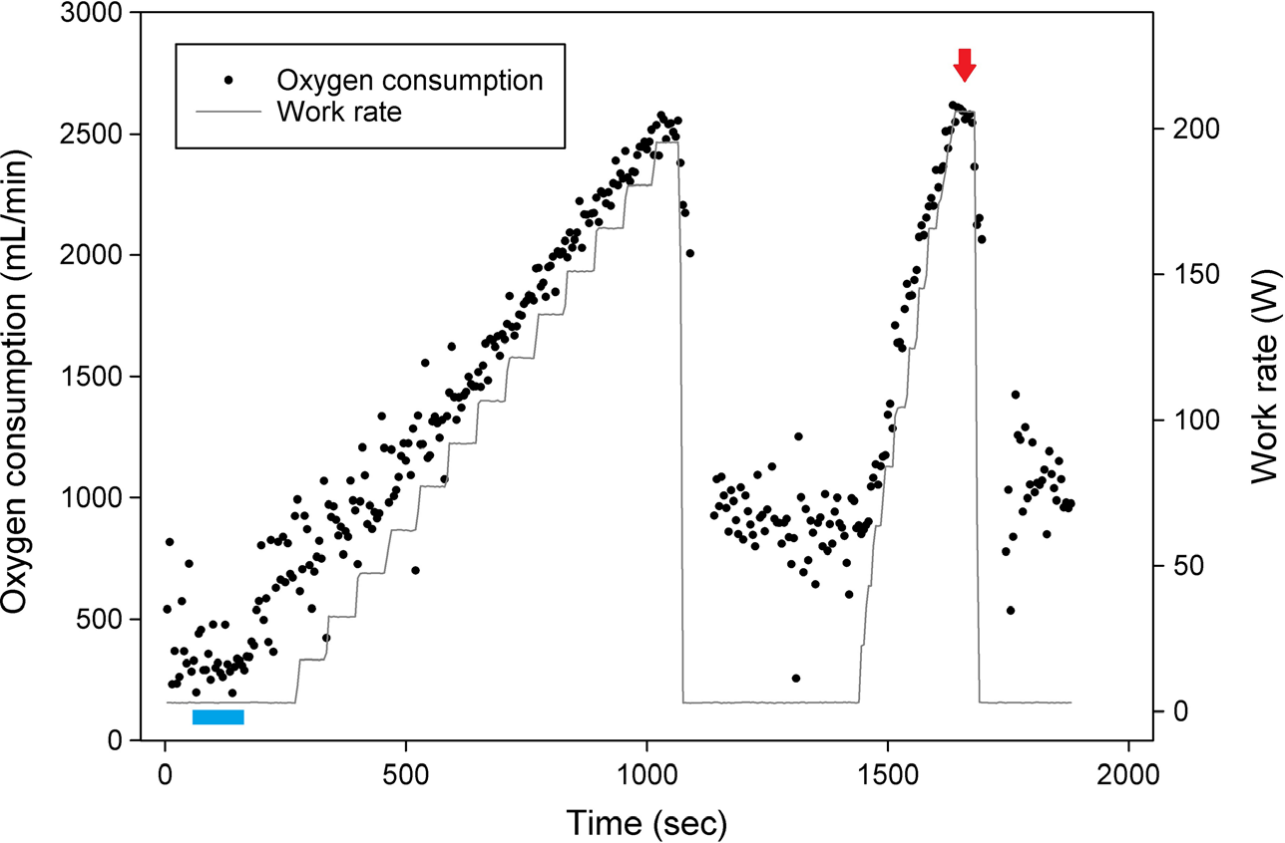
Incremental exercise test protocol and measurement from a representative participant. Maximal oxygen consumption was determined as the average of oxygen consumption values over a 15‐sec interval during the period of volitional exhaustion. The higher value between the two exhaustion periods was chosen, indicated in this case by an arrow. Resting oxygen consumption and carbon dioxide elimination were determined by the average of the 2nd minute period (bar close to the origin).

**Figure 7:**
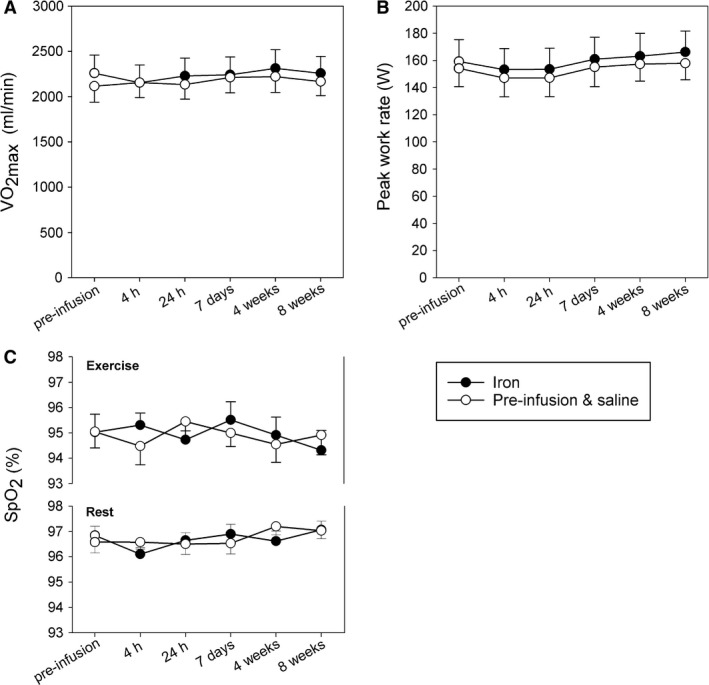
Measurements of maximal oxygen consumption, ${\dot{V}}_{O2max}$, (A), peak work rate (B), and peripheral oxyhemoglobin saturation, SpO_2_, (C) preinfusion (open symbols for both groups) and after infusion for controls (open symbols) and iron group (closed circles). Values of SpO_2_ are given at rest and during maximal exercise. Values are mean ± SEM and did not differ significantly between groups.

Figure 6 shows measurements of oxygen consumption and work rate for a typical volunteer during the incremental exercise test. The responses to heavy exercise during the incremental exercise tests are shown in Figure 7. Linear mixed effects modeling on the postinfusion data using the preinfusion values as covariates and with treatment (iron vs. saline) as a factor found no significant effect of iron administration on either ${\dot{V}}_{O2max}$ or work rate. Successive data points for each subject were treated as repeated measures. Values for peripheral oxyhemoglobin saturation, SpO_2_, at rest and during maximal exercise are also shown in Figure 7. No differences between groups, and no changes from baseline, were observed. Maximal exercise was associated at all times with a fall in SpO_2_ of approximately 1.5–2.0%.

## Discussion

The main finding of this study is that intravenous iron supplementation in older healthy humans leads to a sustained attenuation of the pulmonary artery pressure during moderate air‐breathing (euoxic) exercise. This effect persisted for 8 weeks. We interpret these findings in the context of (1) considering whether the fall in the P_O2_ of venous blood during exercise might act as a stimulus to “hypoxic pulmonary vasoconstriction” even though air‐breathing exercise is usually not regarded as a form of hypoxia; (2) studies finding that administration of vasodilators can reduce pulmonary artery pressure during exercise in hypoxia; (3) observations showing a reduction in hypoxic pulmonary vasoconstriction in humans at rest, induced by administration of iron; and (4) the finding that iron administration can enhance exercise capacity in heart failure. These are discussed in turn below.

### The extent to which euoxic exercise may be regarded as a stimulus for “hypoxic pulmonary vasoconstriction”

Alveolar and systemic arterial levels of oxygenation commonly remain largely unchanged during exercise, as ventilation keeps pace with the elevation in metabolic rate. Mixed venous blood arriving at the lungs, however, is increasingly desaturated as exercise intensity increases (Trinity et al. 2012). Thus, for example, a high work rate in volunteers exercising at sea level was associated with a mixed‐venous P_O2_ below 20 mmHg (Sutton et al. 1988). Studies, using relatively brief experiments in nonhuman species at rest have shown that the stimulus P_O2_ (P_SO2_) for hypoxic pulmonary vasoconstriction may commonly be regarded as a function of both alveolar P_O2_ (P_AO2_) and mixed‐venous P_O2_ (P_VO2_) according to the following relationship (Marshall and Marshall 1983; Marshall et al. 1994a):

(1) $$P_{\mathrm{SO}2}=P_{\mathrm{AO}2}^{0.6}\times P_{\mathrm{VO}2}^{0.4}$$

Interestingly, no study has confirmed this behavior for the human lung, and yet models of human hypoxic pulmonary vasoconstriction have been structured assuming that it is appropriate in humans in health (Marshall et al. 1994c) and disease (Marshall et al. 1994b). Since P_VO2_ falls during exercise, the above equation suggests that exercise may expose the lung to a sufficient reduction in P_SO2_ to activate hypoxic pulmonary vasoconstriction. We explore below what consequences this activation of something usually associated with hypoxic exposure might have if it is actually occurring during euoxic exercise.

### Studies directed at lowering pulmonary artery pressure during exercise in hypoxia

Several studies have addressed the hypotheses that pulmonary artery pressure during exercise can be lowered by pulmonary vasodilators, and that this can lead to an increase in cardiac output and in exercise capacity. In 2004, Ghofrani et al. showed that sildenafil reduced pulmonary artery pressure, and increased cardiac output and peak work rate, both whilst volunteers at low altitude breathed 10% oxygen and whilst at Everest Base Camp at an altitude of 5400 m (Ghofrani et al. 2004). Their volunteers had a median age of 36.5 years. Using another phosphodiesterase‐5 inhibitor, tadalafil, Fischler et al. were unable to show similar changes at low and high (4559 m) altitude in adults known to have particularly vigorous hypoxic pulmonary vasoconstriction, but did find that dexamethasone increased ${\dot{V}}_{O2max}$ significantly, a rise that was associated with a strong trend toward an increase in peak work rate and a lower pulmonary artery pressure (Fischler et al. 2009). Their group had a mean age of 44 years. The authors questioned whether their tadalafil dosing had been adequate. The effect of dexamethasone they observe on ${\dot{V}}_{O2max}$ may be associated with its action as a marked stimulant of ventilation (Liu et al. 2013). Further studies showing no effect of phosphodiesterase‐5 inhibition (using sildenafil) on maximal exercise capacity in acute hypoxia include one at 4000 m equivalent examining volunteers with a mean age 26.8 years (Toro‐Salinas et al. 2016) and another one at 2750 m equivalent recruiting older volunteers with a mean age of 66.5 years (Rodway et al. 2016).

Another approach to lowering right ventricular afterload and increasing exercise capacity in hypoxia has been the use of endothelin receptor inhibition. In acute hypoxia (12% oxygen at sea level) in participants with mean age 29 years, it was reported that the nonselective inhibitor bosentan lowered pulmonary artery pressure and significantly increased maximum work‐rate and ${\dot{V}}_{O2max}$ (Faoro et al. 2009). The Brussels research group went on to assess the effects of the endothelin receptor‐A antagonist sitaxentan both during acute hypoxia (12% oxygen at sea level) and at 5050 m in Nepal, in a group with mean age 35 years (Naeije et al. 2010). Again, both workload and ${\dot{V}}_{O2max}$ were significantly increased by the agents and these changes were related to reductions in right ventricular afterload.

There appears, therefore, to be some evidence that pulmonary vasodilatation during hypoxia can increase exercise capacity possibly by increasing cardiac output. Results may depend upon the degree of hypoxia, agent, dosing, and the age of those studied. Interestingly, we remain uncertain regarding the way in which hypoxic pulmonary vasoconstriction might act to limit cardiac output during exercise. By introducing an increased resistance between the right and left sides of the heart, its action could be via the associated increase in right‐ventricular afterload, via a reduction in the left‐ventricular preload, or a combination of the two. Data suggesting that a reduction in left‐ventricular preload during hypoxic exercise may be an important factor limiting exercise capacity come from the Operation Everest II study, in which eight volunteers spent 40 days in a decompression chamber, in which the pressure was gradually reduced from a “sea‐level” value to that at the summit of Mount Everest (Reeves et al. 1987). Large rises in right‐ventricular afterload were demonstrated by pulmonary artery catheterization at rest and during exercise as the decompression intensified. The study also showed lower filling pressures of the left ventricle during exercise in hypoxia compared with euoxia. In the “sea‐level” conditions, left atrial pressures rose from 6.9 mmHg at rest to 16.4 mmHg at maximum cardiac output. With exercise at “higher altitudes”, the left atrial pressure was found not to rise with exercise above the values at rest (still around 7 mmHg), revealing a contractile ventricle with an output probably limited by poor filling (Reeves et al. 1987).

The studies discussed in this section relate to exercise in hypoxia, either during the breathing of a low percentage of oxygen near sea level, or whilst at altitude. This study was conducted during air‐breathing near sea level. We have shown in the preceding section that the stimulus P_O2_ for pulmonary vasoconstriction may be a function of both alveolar and mixed‐venous P_O2_ values, and that consequently experiments conducted during hypoxic exposure may have a bearing on the euoxic exercise reported here.

### Studies showing a reduction in hypoxic pulmonary vasoconstriction at rest following iron administration

Without having an effect on pulmonary artery pressure in euoxia, an acute infusion of iron has been shown to abolish the slow phase of human hypoxic pulmonary vasoconstriction that develops over 1–8 h of exposure to isocapnic hypoxia at rest (Talbot et al. 2014) and markedly obtund the rise in the acute sensitivity of the pulmonary vasculature to hypoxia (the fast phase of hypoxic pulmonary vasoconstriction) that occurs following such an 8‐h exposure to hypoxia (Smith et al. 2008). Correspondingly, clinical iron deficiency is associated with an enhanced hypoxic pulmonary vasoconstriction response that can be ameliorated by iron infusion (Frise et al. 2016). These studies all suggest that iron supplementation only reduces hypoxic pulmonary vasoconstriction when there is a “priming” period of hypoxia of more than an hour in duration before the effects of iron on hypoxic pulmonary vasoconstriction are measured. This is consistent with the hypothesis that it is transcriptional regulation by the hypoxia‐inducible factor (HIF) pathway that is being manipulated by administering iron, and that this takes time to take effect (Smith et al. 2006, 2008). What remains uncertain is whether a pulmonary vascular response to the lowering of P_O2_ in mixed‐venous blood during euoxic exercise can be attenuated by administering iron.

In this study, exercise was performed for relatively short periods of time not exceeding 1 h, and therefore a salient question is whether iron status can affect pulmonary vascular responses to periods of desaturation of mixed‐venous blood lasting less than 1 h. The human pulmonary vascular response to eucapnic alveolar hypoxia in people at rest starts with an abrupt acute vasoconstriction (a fast phase) occurring over a few (~5) minutes. If the hypoxia is maintained, this constriction begins to intensify after about 40 min (Talbot et al. 2005), and continues to increase over the periods of 2–8 h (a slow phase) that have been studied in various laboratory protocols (Dorrington et al. 1997; Balanos et al. 2002; Talbot et al. 2014). Prior infusion of iron has not been found to change the magnitude of the fast phase, often measured at ~1 h, but does have a marked effect in diminishing or abolishing the subsequent slow phase, over 2–8 h (Talbot et al. 2014; Bart et al. 2016; Frise et al. 2016). This blunting of the slow phase of hypoxic pulmonary vasoconstriction has now been shown to persist for at least as long as 43 days after a single infusion of iron, with the 1‐h fast‐phase response remaining unaffected throughout this period (Bart et al. 2016).

The accumulated evidence therefore suggests that prior iron administration in the resting individual has the capability to (1) reduce the slow phase of hypoxic pulmonary vasoconstriction and (2) reduce the exaggeration of the fast phase of hypoxic pulmonary vasoconstriction that is induced by priming with a sustained period (~8 h) of hypoxia. We lack evidence that iron administration can influence the fast phase of hypoxic pulmonary vasoconstriction unless this priming takes place.

### Possible explanations for the finding of a reduced SPAP during exercise following iron

The observation in this study (Fig. 4) that SPAP during exercise is decreased from 3 h following an iron infusion may therefore provide evidence that: (1) the model represented by the above equation for P_SO2_ is valid for humans, (2) the intermittent lowering of mixed‐venous P_O2_ in small bouts of exercise occurring in normal daily life constitute a cumulative priming stimulus for the pulmonary vasculature yielding: (3) a reduction in an acute pulmonary vascular response to a brief (20‐min) bout of exercise in those volunteers who received iron. If there is no sensitivity of the human pulmonary vasculature to a lowering of P_VO2_ of the sort expressed in the equation, it is difficult to understand why iron has the capacity to reduce the rise in SPAP occurring during exercise.

An alternative explanation could be that improved left‐ventricular function following iron administration could permit the same level of exercise with a lower left‐sided preload. If that is the case, a fall in filling pressure of ~3 mmHg would represent a substantial change in inotropic state.

### Enhancement of exercise capacity following iron administration in patients with cardiopulmonary disease

The administration of intravenous iron to nonanemic iron‐deficient patients with heart failure has been found to lead to an improvement in exercise capacity measured by the 6‐min walk test in two similar studies over 6 months (Anker et al. 2009) and 12 months (Ponikowski et al. 2015). Similar results have emerged from studies on patients with pulmonary arterial hypertension (Viethen et al. 2014; Ruiter et al. 2015). One possible contributory mechanism is the finding that iron deficiency is associated with much more marked hypoxic pulmonary vasoconstriction, and that this abnormality can be corrected by administration of intravenous iron (Frise et al. 2016). Cardiopulmonary measurements in exercise were not obtained in the heart‐failure and pulmonary arterial hypertension studies, so we remain ignorant of whether a reduction in right ventricular afterload or an increase in left ventricular preload may have assisted walking in the exercise tests. This study was conducted in iron‐replete healthy participants of a similar age; nevertheless its finding of a reduction in pulmonary artery pressure during moderate cycling exercise tentatively suggests a mechanism whereby exercise capacity may have been improved in the patients in clinical studies. If this turns out to be the case, it would be analogous to the enhancement of exercise achieved in some studies of healthy human volunteers exposed to hypoxia, such as that using sildenafil at Everest Base Camp (Ghofrani et al. 2004). It may be the case that, whilst the small fall in pulmonary artery pressure measured in this study has no impact on maximal exercise capacity in our healthy volunteers, it could be sufficient to affect exercise capacity in a patient with impaired myocardial function.

As noted earlier, another possible mechanism whereby pulmonary artery pressure is lowered in exercise by iron supplementation is an improvement in left ventricular function. Such an effect was suggested by a study of patients with heart failure and iron deficiency in the setting of chronic renal failure (Toblli et al. 2015). This could lead to a lowering of left‐sided filling pressure for the same level of cardiac output. This would result in a lower pulmonary artery pressure without any change in the pressure drop across the pulmonary circulation.

## Conclusions

Iron administration appears to act as a pulmonary vasodilator selectively during exercise in the older healthy human. The effect is maintained over many weeks after a single dose of iron. A body of literature links this observation with the inhibition by iron of vasoconstriction in the lung, via the HIF transcription factor pathway. Elevated SPAP can be implicated as a factor limiting exercise capacity, particularly in the aging population (Kovacs et al. 2009a), and in those with underlying lung or connective tissue disease (Kovacs et al. 2009b). Iron has already been shown to improve exercise tolerance in patients with heart failure (Anker et al. 2009), and this may be due to effects on the pulmonary vasculature.

## Conflict of Interest

None declared.
